# Supplementary material for: Long-Term Nightshift Work and Breast Cancer Risk: An Updated Systematic Review and Meta-Analysis with Special Attention to Menopausal Status and to Recent Nightshift Work
Source: Cancers (Basel). 2021 Nov 26;13(23):5952. doi: 10.3390/cancers13235952 (PMC8657038; doi:10.3390/cancers13235952)
Supplement: Supplementary file 1 [file cancers-13-05952-s001.zip › cancers-1430620-supplementary.pdf]

Supplementary materials: Long-Term Nightshift Work and Breast Cancer Risk: An Updated Systematic Review and Meta-Analysis with Special Attention to Menopausal Status and to Recent Nightshift Work

Christine Schwarz, Ana María Pedraza-Flechas, Roberto Pastor Barriuso, Virginia Lope, Nerea Fernández de Larrea, José Juan Jiménez-Moleón, Marina Pollán and Beatriz Pérez-Gómez

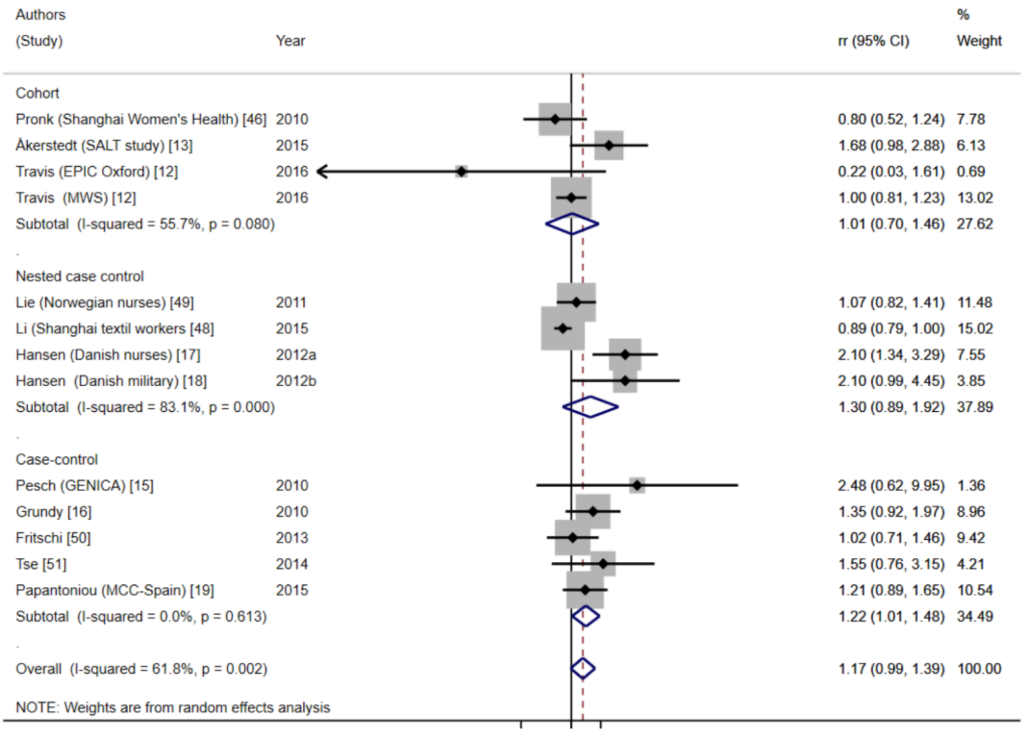

Figure S1. Analysis excluding five studies with uncertain exposure definitions.

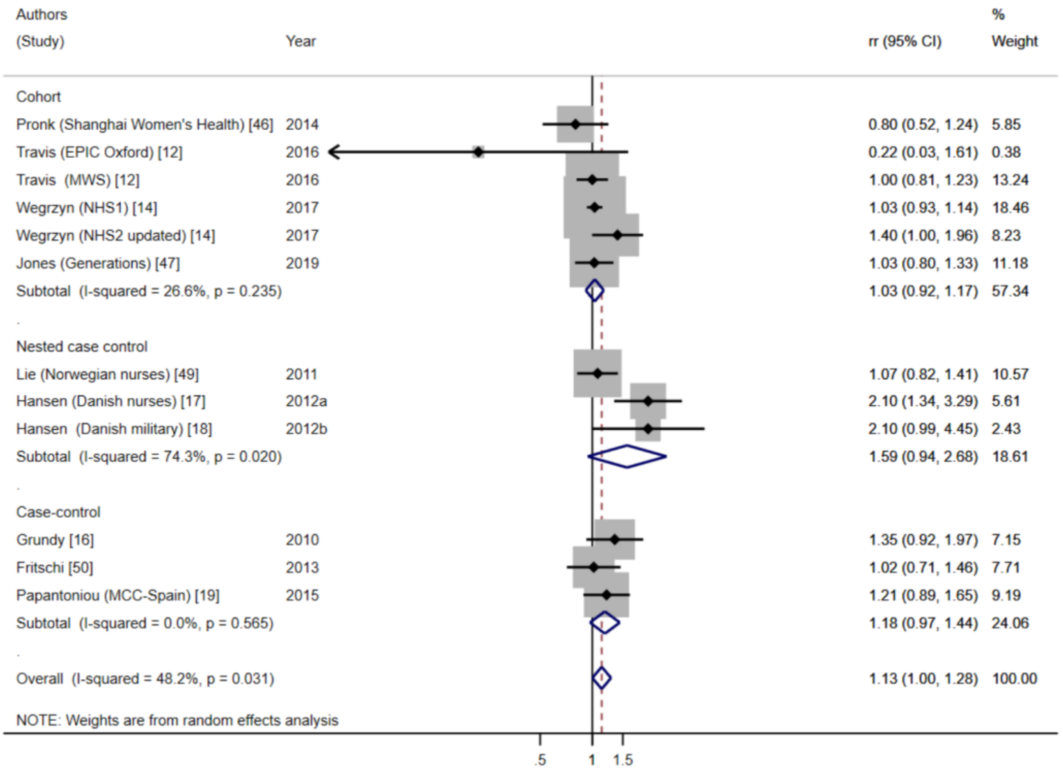

Figure S2. Analysis of long-term NSW and BC restricted to those studies that reached at least seven. points in the Newcastle Ottawa Scale (NOS ≥ 7).

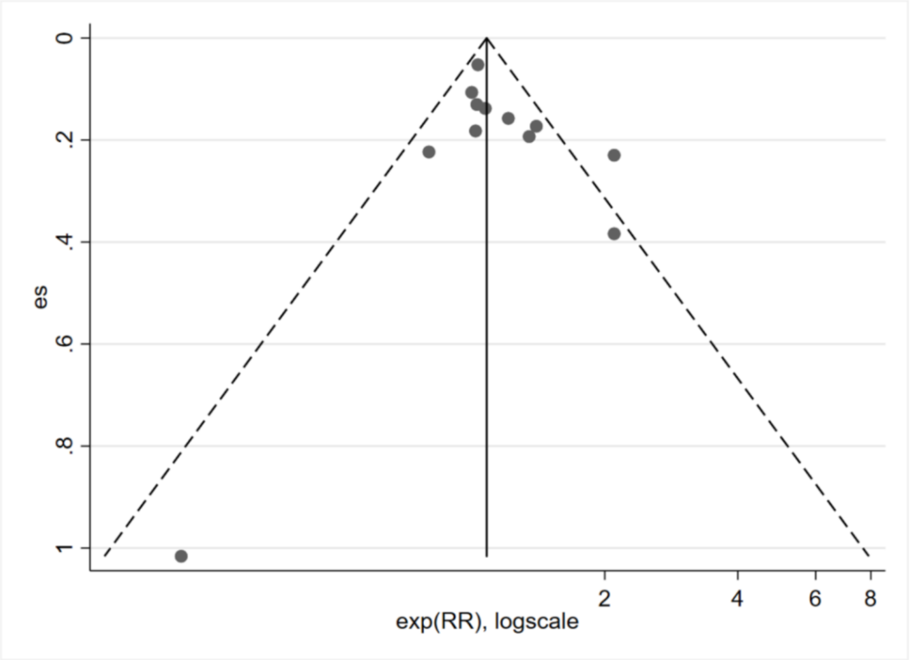

Figure S3. Funnel plot limited to studies of high quality (NOS ≥ 7).

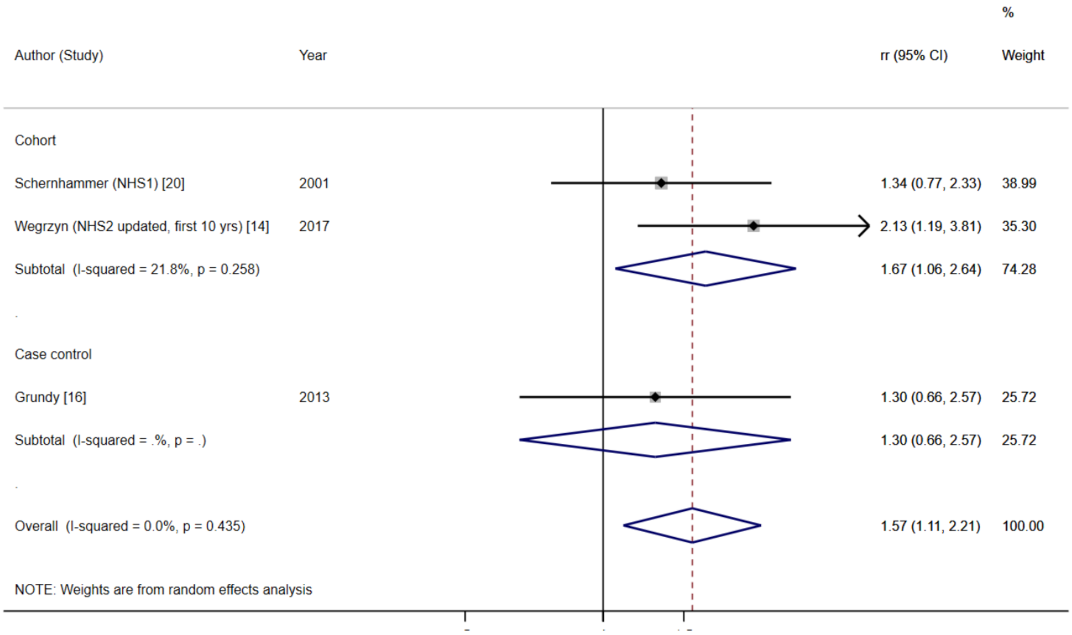

Figure S4. Analysis of long-term NSW and premenopausal BC limited to studies of high quality (NOS ≥ 7).

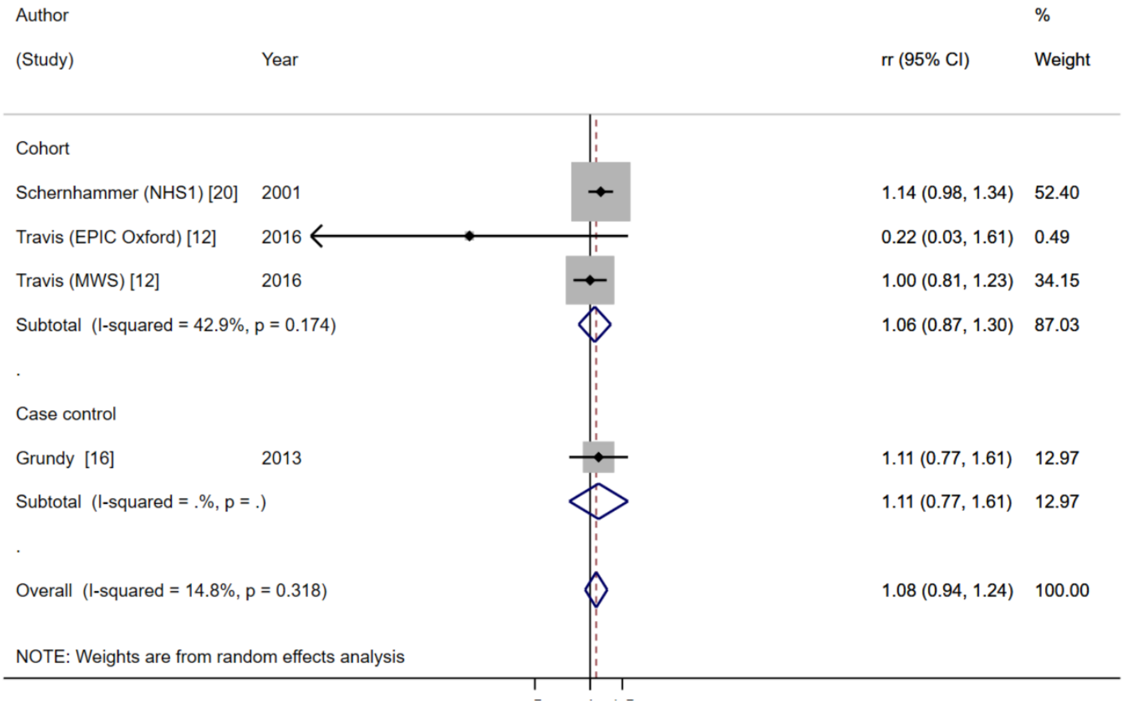

Figure S5. Analysis of long-term NSW and postmenopausal BC limited to studies of high quality (NOS ≥ 7).

Table S1. Results of the risk of bias assessment by the Newcastle-Ottawa Scale.

Cohort Studies	I. Representativeness/Selection				II.Comparability	III. Outcome			
Author, study, publication date	I.1. Exposed cohort	I.2 Non exposed cohort from the same community	I.3 Exposure Ascertainment	I.4 Outcome not present at start	II. Control of covariables	III.1 Outcome Assessment	III.2. Follow-up length (>15 yrs)	III.3. Follow-up adequacy (<15% of difference in lost to follow-up)	Q
Wegrzyn (2017) NHS I [14]		✡	✡	✡	✡✡	✡	✡	✡	8
Wegrzyn (2017) NHS II [14]		✡	✡	✡	✡✡	✡	✡	✡	8
Pronk (2010) SHWS [46]	✡	✡	✡	✡	✡	✡	✡		7
Koppes (2014) Dutch Labor Force [22]	✡	✡		✡		✡			4
Åkerstedt (2015) SALT [13]	✡	✡	✡	✡	✡	✡			6
Travis (2016) EPIC-Oxford [12]	✡	✡	✡	✡	✡✡	✡			7
Travis (2016) MWS UK [12]	✡	✡	✡	✡	✡✡	✡			7
Jones (2019) Generations Study [47]	✡	✡	✡	✡	✡✡	✡			7
**Nested case-control and case control studies**	**I. Selection**				**II. Comparability**	**III. Ascertainment of exposure**			
**Author, study, publication date**	**I.1 Adequate case definition**	**I.2 Representativeness of the cases**	**I.3 Controls selection**	**I.4 Controls definition**	**II. Control of covariables**	**III.1 Exposure ascertainment**	**III.2 Same method for cases & controls**	**III.3 Non-response-rate (<15% of difference)**	**Q**
**Nested case- control studies**									
Lie (2006) Norwegian nurses [21]	✡		✡	✡			✡	✡	5
Lie (2011) Norwegian nurses [49]	✡		✡	✡	✡	✡	✡	✡	7
Hansen (2012a) Danish nurses [17]	✡		✡	✡	✡✡		✡	✡	7
Hansen (2012b) Danish military [18]	✡		✡	✡	✡✡	✡	✡	✡	8
Li (2015) Shanghai textile worker [48]	✡		✡	✡			✡	✡	5
**Case- control studies**									
Pesch (2010) GENICA [15]	✡	✡	✡	✡	✡		✡		6
Fritschi (2013) BCEES [50]	✡	✡	✡	✡	✡	✡	✡		7
Grundy (2013) [16]	✡	✡	✡	✡	✡✡		✡	✡	8
Tse (2014) [51,55]	✡	✡		✡	✡		✡	✡	6
Papantoniou (2015) MCC [19]	✡	✡	✡	✡	✡✡		✡		7
